# Prevalence, Features and Degree of Association of Oral Lesions in COVID-19: A Systematic Review of Systematic Reviews

**DOI:** 10.3390/ijerph19127486

**Published:** 2022-06-18

**Authors:** Federica Di Spirito, Alfredo Iandolo, Alessandra Amato, Mario Caggiano, Annunziata Raimondo, Serena Lembo, Stefano Martina

**Affiliations:** Department of Medicine, Surgery and Dentistry, University of Salerno, 84084 Salerno, Italy; aiandolo@unisa.it (A.I.); aamato@unisa.it (A.A.); macaggiano@unisa.it (M.C.); araimondo@unisa.it (A.R.); slembo@unisa.it (S.L.); smartina@unisa.it (S.M.)

**Keywords:** COVID-19, coronavirus disease 2019, oral lesions, oral manifestations, SARS-CoV-2

## Abstract

Regardless of rapidly emerging findings on oral lesions described in adult SARS-CoV-2-positive subjects, the evidence level remains quite low and rather contrasting; therefore, the present systematic review of systematic reviews primarily aims to point out the overall prevalence of diagnosed cases. Secondary aims are to estimate the degree of association between oral lesions and SARS-CoV-2 infection and to grade, based on the reported frequency, the primary oral lesions, with related clinical presentations and microscopic features, in relation to COVID-19 forms. A study protocol compliant with the PRISMA statement was developed. Twelve studies were included, reporting highly heterogeneous and incomplete findings, thus precluding a meta-analysis. Further studies should be conducted to assess the overall prevalence of cases diagnosed with oral lesions among adult SARS-CoV-2-positive subjects, especially considering novel viral variants, and to determine their degree of association with SARS-CoV-2 infection and COVID-19 forms. Moreover, the reported findings noticed the need to evaluate the putative role both of SARS-CoV-2 in oral lesions genesis and of periodontitis and periodontal microbiome in COVID-19 worsening and re-activations. Deeper insights into oral lesions in adult SARS-CoV-2-positive subjects could enhance the comprehension of illness pathogenesis, natural history and clinical presentation, thus improving the preparedness of health professionals in the inter-disciplinary management of COVID-19.

## 1. Introduction

The most common manifestations of COVID-19 comprise asthenia, headache, fever, hyposmia, oropharyngeal inflammation, dysgeusia, dry cough, dyspnea, vomiting, abdominal pain and diarrhea [1,2]. Further enriching the compound clinical presentation of the disease, muco-cutaneous manifestations, resembling varicella and measles rushes, as well as Erythema Multiforme lesions, urticaria and petechiae [3,4] have also been reported in SARS-CoV-2-positive subjects.

Such multi-system involvement characterizing COVID-19 may be attributable to the wide topographic distribution of the viral Angiotensin Converting Enzyme 2 (ACE2) binding receptor, mediating cell invasion, which has been detected in upper and lower respiratory apparatus; gastrointestinal tract, including oral epitheliocytes; exocrine glands, along with salivary ones; cardiovascular and genitourinary systems; skeletal muscles and skin [5,6,7]. Consistently, multiple case reports and series, as well as letters to the Editor and comments [8,9,10,11], have later described several lesions of the oral mucosa potentially ascribable to SARS-CoV-2 infection or observed in patients with COVID-19, clearly highlighting heterogeneous macroscopic features and uncertain prevalence.

Furthermore, regardless of rapidly emerging findings, the evidence level seems to remain quite low and rather contrasting; therefore, the present systematic review of systematic reviews primarily aims to synthesize, integrate and update currently available data on the overall prevalence of cases diagnosed with oral lesions among adult SARS-CoV-2-positive subjects. Secondary aims of the study are to estimate the degree of association between oral lesions and SARS-CoV-2 infection [12] and to grade, based on the reported frequency, the primary oral lesions, with related clinical presentations and microscopic features, in relation to COVID-19 forms [13].

## 2. Materials and Methods

The presently applied study protocol was developed before literature search and data analysis in compliance with the Preferred Reporting Items for Systematic Reviews and Meta-Analyses (PRISMA) statement [14] focusing the research questions on: prevalence, clinical presentation and histopathology of oral lesions diagnosed in adult SARS-CoV-2-positive subjects [15].

### 2.1. Search Strategy

Systematic reviews with or without meta-analysis on oral lesions observed in SARS-CoV-2-positive patients were electronically searched till 7 May 2022, across Scopus, MEDLINE/PubMed and BioMed Central databases, using review, systematic reviews and no filters, respectively, as well as the PROSPERO register, by two independent reviewers (F.D.S. and A.I.), without language restrictions and using the following suitable key words, appropriately combined by Boolean operators:

1. COVID-19 OR SARS-CoV-2 OR Coronavirus disease 2019 OR novel coronavirus
2. AND oral lesions OR oral manifestations OR oral signs
3. AND Systematic review OR meta-analysis.

### 2.2. Study Selection and Eligibility Criteria

The collected citations were recorded; then, the duplicates were eliminated, and the obtained titles and abstracts were independently screened by two reviewers (F.D.S. and A.I.). The fulltexts of potentially relevant papers and ambiguous abstracts were independently reviewed by the same authors (F.D.S. and A.I.), who solved disagreements through discussion and consensus, by involving a third author (S.M.) if necessary. An additional manual search for systematic reviews with or without meta-analysis was also conducted on the reference list of eligible articles; pertinent titles and abstract were screened, and the fulltexts were reviewed as already described. The contacting of study authors was conducted in case of missing fulltexts.

No restrictions were applied during literature search concerning: status, date, language of the publications; study design; sample size, comorbidities and COVID-19 severity of the investigated population. The exclusion criteria were: participants aged < 18 years old; normal variations [16]; likely pre-existing and self-reported lesions of the oral mucosa; oral lesions following COVID-19 vaccination; smell and taste dysfunction.

### 2.3. Data Extraction

Data were independently extracted on a standardized data extraction form by two reviewers (F.D.S. and S.M.), who reached consensus by discussion, also involving a third author (A.I.) if needed.

From each of the systematic reviews with or without meta-analysis included in the present study, the following data meeting eligibility criteria were recorded, when available: first author, year, journal, date of publication, funding, quality of the systematic reviews included in the present study; design and number of reported studies; sample size, gender ratio, mean age and COVID-19 ongoing treatment and severity, classified as mild/moderate or severe/hospitalized, as per Amorim Dos Santos et al. [17], of the investigated population; total number or prevalence of subjects diagnosed with oral lesions and defined as “cases”; clinical presentation and microscopic features of the oral lesions described.

### 2.4. Quality Assessment and Data Synthesis

The Methodological Quality of Systematic Reviews (AMSTAR) 2 tool [18], accessed online (https://amstar.ca) on 14 May 2022, the assessment of the quality of the systematic reviews of both randomized and/or non-randomized studies included in the present study was employed.

### 2.5. Synthesis Methods

Descriptive statistical analyses were conducted using Statistical Package for the Social Sciences (SPSS version 25.0; Armonk, NY, USA Microsoft Excel software 2019; Microsoft Corporation, Redmond, WA, USA).

## 3. Results

### 3.1. Study Selection

In total, 351 potentially relevant titles/abstract of systematic reviews were obtained from the electronic search, 15 of which were from MEDLINE/PubMed, 12 from Scopus and 322 from the BioMed Central database, along with 2 from the PROSPERO register. A total of 14 duplicates were removed along with 1 ongoing not-published review from the PROSPERO register. After title/abstract screening, three records, one evaluating the risk of virus contamination during minimally invasive surgery [19], one limiting the investigation to oral manifestations in children [20] and one reporting lesions observed following COVID-19 vaccination [21], were excluded. Finally, 12 studies [22,23,24,25,26,27,28,29,30,31,32,33] compliant with the eligibility criteria were included in the present systematic review of systematic reviews.

**Figure 1:**
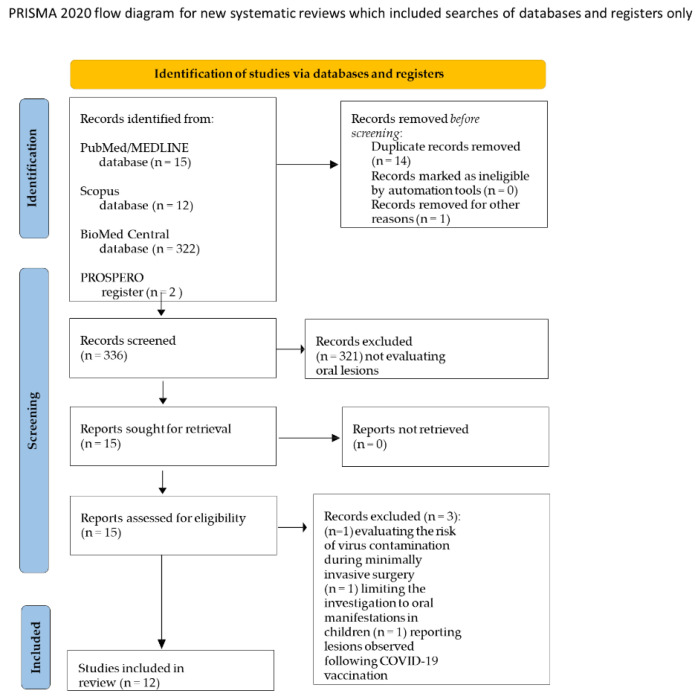
PRISMA flow chart.

The study-selection flowchart is illustrated in Figure 1.

### 3.2. Studies’ Characteristics and Qualitative Synthesis

Out of the twelve systematic reviews included in the present study, detailed in Table 1, all resulted to have been published in the English language between 11 September 2020 and 27 April 2022; the full texts were all available, and two authors [30,32] declared funding. Two systematic reviews exclusively comprised case reports and case series [27,32], with one also including letters to the Editor and comments [33], whilst the remaining nine [22,23,24,25,26,28,29,30,31] also considered observational cross-sectional and/or retrospective and/or prospective studies. Three systematic reviews included a meta-analysis [23,28,30], and all resulted to be of a critically low quality, except for Doceda et al.’s [26], Nijakowski et al.’s [28] and Orilisi et al.’s [29] studies, characterized by low quality, as per the AMSTAR2 tool assessment.

In compliance with the eligibility criteria, the data reported in a total of 176 studies, 65 observational studies and 108 case reports, as well as case series, letters to the Editor and comments, included in the presently considered systematic reviews were extracted and recorded.

The findings from a total of 11,717 SARS-CoV-2-positive subjects ≥ 18 years old were obtained. The mean age of the investigated population was retrieved or presently computed by reported data from six systematic reviews [22,24,28,29,32,33], generally involving a population of 3288 subjects, between 40.5 and 56.6 years old, with a mean of 50.0 years of age (8429 study participants without reported age). The gender ratio of the investigated population was specified in six studies [22,24,26,28,29,32], accounting for 4925 subjects, 3156 males and 1769 females (6792 study participants without reported gender). COVID-19 illness severity was defined, differentiating hospitalized and non-hospitalized subjects, in six systematic reviews [22,25,26,28,29,30]; in the systematic review conducted by Erbaş et al., 2022 [27], 76.1% of the overall population was declared to be hospitalized, but it was not definable from the reported findings if the involved subjects were ≥18 years old; therefore, such cases were not presently considered. Ongoing COVID-19 treatment, instead, was described in three studies [26,28,32], generally reporting the administration of paracetamol, dexamethasone, chloroquine, antibiotics and antivirals.

**Figure 2:**
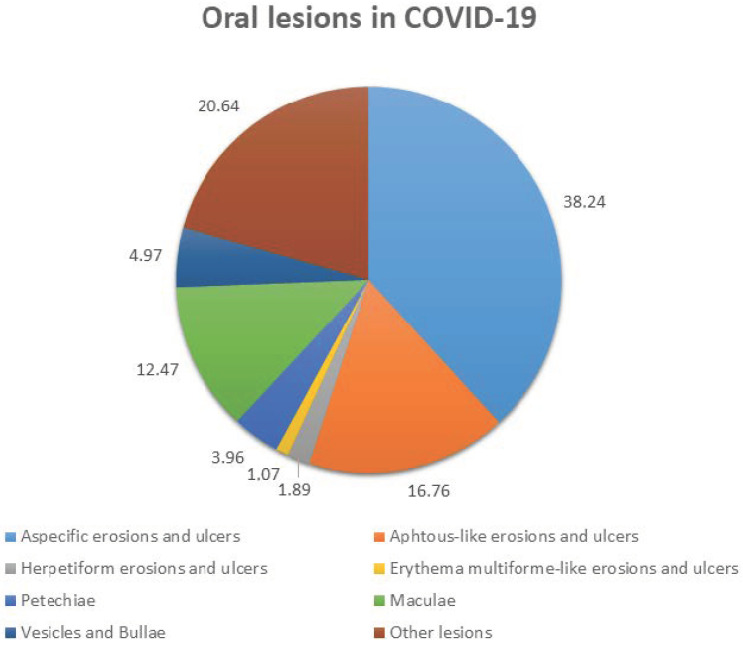
Reported frequency of oral lesions in COVID-19.

Among the SARS-CoV-2-positive subjects ≥ 18 years old considered in the twelve systematic reviews, 13.54% were diagnosed with oral lesions; the cases’ mean age and gender ratio could not be calculated due to the paucity of the reported data. Erosions and ulcers were reported in 48.96% of the cases, with no specific characteristics (38.24%) or described as aphthous-like (16.76%), erythema multiforme-like (1.89%) and herpetiform (1.07%), as illustrated in Figure 2. White plaques were found in 0.25% of cases with oral lesions, and no red plaques were recorded, while vesicles and bullae accounted for the 4.97% of the diagnosed cases (Figure 2). Maculae (12.47%) and petechiae (3.96%) altogether resulted to be described in 16.44% of the cases (Figure 2). Oral candidiasis (10.74%), lichenoid (4.63%) and hemorrhagic (2.73%) lesions, as well as necrotizing periodontal disease (1.64%) and desquamative gingivitis (0.90%), were also reported. The affected locations were defined in six systematic reviews [22,24,26,27,28,32], reporting a total of 546 cases, and the tongue was found to be the most frequently affected site (18.58%), followed by buccal and labial (13.73%) and palatal (2.70%) mucosa, gingiva (2.83%) and diffuse lesions (2.33%). Microscopic features were detailed for two cases in Amorim Dos Santos et al.’s [22] and for five cases in Silveira et al.’s [32] systematic reviews; two of the seven described cases were reported in both studies and all five histopathological findings concerned oral ulcers.

## 4. Discussion

A large body of evidence has described lesions of the oral mucosa, potentially ascribable to SARS-CoV-2 infection or observed in patients with COVID-19, clearly highlighting heterogeneous macroscopic features and uncertain prevalence [8,9,10,11]. Therefore, the present systematic review of systematic reviews aims, primarily, to point out the overall prevalence of cases diagnosed with oral lesions among adult SARS-CoV-2-positive subjects and to, secondarily, estimate the degree of association between oral lesions and SARS-CoV-2 infection [12] and grade, based on the reported frequency, the primary oral lesions, with related clinical presentations and microscopic features, in relation to COVID-19 forms [13].

Out of the twelve systematic reviews currently considered, none included trials, reliably not conducted at the time, and seven also comprised a total of 65 observational studies. All of the systematic reviews included in the present study analyzed data from cumulatively 108 case reports and series, letters to the Editor and comments that, despite the intrinsic low level of evidence, accurately describe both the oral features and systemic conditions of the cases.

Since data from studies included in the systematic reviews but not meeting eligibility criteria were not considered, findings concerning younger subjects were excluded from the current analysis. In addition, the average age of the cases diagnosed with oral lesions was only reported in six out of twelve systematic reviews [24,25,28,29,32,33], revealing a mean age of 50.0 and not allowing a complete computation. Similarly, since appropriate data could only be extracted from six out of twelve systematic reviews [22,24,26,28,29,32], showing a male–female ratio of 3156/1769, a precise estimate could not be carried out, not permitting a comparison with Nuno-Gonzalez et al.’s [34] results, pointing out a slightly higher prevalence of oral lesions in females potentially related to the influence of sex hormones on SARS-CoV-2 pathophysiology [35]. Severely incomplete data were also found concerning COVID-19 severity in the investigated populations, currently classified as mild/moderate or severe/hospitalized, as per Amorim Dos Santos et al. [22]. Consequently, the degree of association of oral lesions with SARS-CoV-2 infection [12] could not be determinately estimated, and the primary lesions, with related clinical presentations and microscopic features, could not be definitively graded in relation to COVID-19 forms [13]. Similarly, largely deficient data were acquired concerning ongoing COVID-19 treatments, potentially responsible of oral adverse reactions. Indeed, oral lesions, enriching the multi-systemic phenomenology of the illness, have been hypothesized to be potentially ascribable to the direct cytopathic effect of SARS-CoV-2, with the viral invasion of the oral epitheliocytes, mediated by ACE2 receptors [5,6] or attributable to the pathophysiology of SARS-CoV-2 infection; therefore, they could manifest as an indirect epiphenomenon of the immune–inflammatory reaction against viral antigens [36]. Moreover, the reported oral lesions were proposed to possibly represent the clinical expression of secondary immunity impairment and related co-infections, as well as adverse reactions to pharmacological therapies and iatrogenic injuries related to COVID-19 treatment [32,37,38].

However, regardless of the proposed pathogenesis of oral lesions occurring in COVID-19, which goes beyond the scope of the present study, the overall prevalence of cases diagnosed with oral lesions in SARS-CoV-2-positive subjects ≥ 18 years of age is still debated. Such prevalence may be very difficult to be determined and, conceivably, under-evaluated as a result of life-threatening conditions and supplementary breathing bias use in severe and critical COVID-19 patients, as well as domiciliary care in mild illness, beyond the particularly defensive personal protective equipment limiting photographic documentation. The total cases diagnosed with oral lesions accounted for 13.54% of the population analyzed in the twelve systematic reviews. Noteworthy, it may be hypothesized that the presently computed prevalence could be affected by the high number of case reports and series considered in the systematic reviews, thus potentially resulting overestimated, although Fidan et al.’s [39] and Elamrousi et al.’s [40] studies reported prevalence values as high as 65.5% and 90.3%. Such discrepancies may be explained by the exclusion from the present analysis of oral lesions self-diagnosed by means of questionnaires, which were included, instead, in Qui et al.’s review [30], of normal variations, such as fissured and geographic tongue, and of likely pre-existing conditions and diseases, principally represented by Oral Lichen Planus, which were also recorded (Table 1).

The retrieved findings revealed extremely heterogeneous denominations of primary oral lesions, probably referable to the fact that the cases were mainly diagnosed by internists, anesthesiologists and dermatologists, rather than oral medicine consultants and dentists, especially during the first peak of the pandemic and the related suspension of non-urgent care. Given these considerations, the authors categorized the reported primary oral lesions as aphthous-like and/or erythema multiforme-like and/or herpetiform erosions and ulcers, white and/or red plaques, and vesicles and bullae, attempting to reduce heterogeneity in reporting forms and suggesting the use of such common denominations in future research. In addition, a high frequency of cases with polymorphous and complex intra- and peri-oral macroscopic pictures clearly emerged, making extremely complex the identification of the underlying primary lesions (Table 1). However, the most frequently reported primary oral lesions resulted to be erosion and ulcers, found in 48.96% of diagnosed cases; in more detail, no specific characteristics were delineated for 38.24% of such lesions, while aphthous-like, herpetiform and erythema multiforme-like appearances were described in 16.76%, 1.89% and 1.07% of the overall erosions and ulcers recorded, respectively (Figure 2). Similarly, Fidan et al. [39] described erosions and ulcers as the most frequent primary oral lesions in SARS-CoV-2-positive subjects and estimated a prevalence of 39.7% of aphthous-like ones. Second for reported frequency were maculae (12.47%), mostly depicted as erythema, and petechiae (3.96%), representing 16.44% of the primary oral lesions, followed by vesicles and bullae, mainly diffuse, and plaques, exclusively white, accounting for 4.97% and 0.25% of the reported oral lesions (Figure 2). Such primary oral lesions could not be analyzed in relation to the most frequently affected intra-oral site due to the incompleteness of findings, although tongue, buccal and palatal mucosa, gingiva and lip resulted to be involved in a descending order, also in accordance with Iranmanesh et al. [37], representing the tongue and labial mucosa as the most common affected locations. Particularly relevant would have also been identifying the timing of the appearance of the described oral lesions, reported only by Orilisi et al. [29]; this has been previously proposed to be a potential indicator both of COVID-19 onset, as established for taste and smell alterations, and illness worsening [1,2,8,9,10,11]. Moreover, broadening the knowledge of the natural history of the disease, also including the expected timing of the appearance of oral lesions in COVID-19, may bring a two-fold effect: on one hand, it may aid in categorizing lesions into early ones, potentially attributable to the viral cytopathic effect, and late ones, detected after the start of therapy and potentially associated with it [41], while, on the other hand, it may improve oral and dental care provision planning, as part of the interdisciplinary management of patients with COVID-19.

At the current state of knowledge, since the histopathological features of oral mucosal lesions were only reported in few cases [17,32,41] diagnosed with oral erosions or ulcers by mostly describing similar non-specific pictures (Table 1), no definitive evidence on the microscopic appearance of the oral lesions most frequently observed in adult SARS-Cov-2-positive subjects has been achieved. However, the most commonly reported histopathological feature for such erosive and ulcerative oral lesions is the vacuolization of oral epithelial cells, which, similarly to the microscopic alterations observed in other infections by epitheliotropic viruses, including those belonging to the *Herpesviridae* family, could constitute the epiphenomenon of the direct cytopathic effect operated by SARS-CoV-2. The latter hypothesis would be validated by the contextual detection of the virus within oral epithelial layers by in situ hybridization or immunohistochemistry, which was conducted, at the current state of evidence, only by Soares et al. [42]. Necrotic phenomena, leukocytosis and Langerhans cell activation were also described within the oral epithelium [22,32,41]. In the underlying connective tissue, similar to cutaneous and pulmonary biopsies, multiple micro-thrombi with consequent partial or total occlusion of small-and medium-caliber vesselswere also described, along with massive inflammatory cell infiltration at peri-vascular and peri-glandular sites, as well as with a band-like lichenoid distribution, and reactive vascular hyperplasia and peri-vascular fibrosis [22,32,41].

In addition to primary oral lesions, desquamative gingivitis (0.63%), lichenoid (4.2%) and hemorrhagic (2.3%) lesions were also reported, as well as oral candidiasis (5.4%) and necrotizing periodontal disease (1.27%), probably attributable to immune impairment. Besides the periodontal necrotizing lesions, also those from chronic periodontitis were related to the COVID-19, and the most severe stages of periodontitis were associated with higher rates of hospitalization, need of ventilation and mortality [43]. The authors of this systematic review conclude that a periodontal state assessment could help identify those at risk, recognizing as statistically significant and common risk factors both COVID-19 and periodontitis in diabetes mellitus and cardiovascular diseases [43,44]. Indeed, it has been hypothesized that periodontitis, poor oral hygiene and periodontal microbiome may themselves constitute risk factors for complications from COVID-19 and the worsening of illness forms [26,45]. It may be consequently proposed that instructions and motivation for oral hygiene and, above all, active periodontal treatment should be systematically integrated into inter-disciplinary management in subjects with mild and moderate COVID-19, and where possible, in severe cases, especially in those with diabetes and cardiovascular disease. Furthermore, considering periodontal pockets as possible reservoirs of SARS-CoV-2, as already amply demonstrated for herpesviruses, including herpes simplex, Epstein-Barr virus andhuman cytomegalovirus [46], it may be hypothesized that periodontal treatment and oral antisepsis with chlorhexidine and hydrogen peroxide, by controlling suspected periodontal pathogens and viral microbial load, may reduce the risk of re-infection in COVID-19 healed subjects [11,47].

Despite the very inclusive eligibility criteria applied, only few and poor-quality systematic reviews could be retrieved. Moreover, high heterogeneous and incomplete data were extracted, precluding the possibility of conducting a meta-analysis and achieving definitive results, which may represent the main limitation of the study. However, the present study may be the first to synthesize findings from available systematic reviews and aim to grade, based on the reported frequency, the oral primary lesions, with clinical presentations and microscopic features, and to estimate their degree of association with SARS-CoV-2 infection [12] and with COVID-19 severity [13].

Further studies with a higher evidence level should be conducted to accurately describe and assess the prevalence of cases diagnosed with oral lesions among adult SARS-CoV-2-positive subjects, especially considering novel variants and occurring following vaccine administration. Additional investigations may also aid in grading the frequency of primary oral lesions with related clinical presentations and microscopic features and in determining their degree of association with SARS-CoV-2 infection [12] and with COVID-19 forms [13]. Furthermore, the putative role of SARS-CoV-2 in oral lesion genesis, on the one hand, and the supposed contribution of periodontitis and periodontal microbiome, seemingly interconnected with the gut–lung axis [45], in COVID-19 worsening and re-activations, on the other hand, should be evaluated.

Deeper insights into oral lesions in adult SARS-CoV-2-positive subjects, which may behypothetically attributable to the direct cytopathic effect of SARS-CoV-2 or to the indirect effect of the immune–inflammatory reactions occurring in the course of the disease or may be somehow related to COVID-19 pharmacological therapy and treatment procedures, could enhance the comprehension of illness pathogenesis, thus improving the preparedness of health professionals, including oral healthcare workers [4,48,49,50,51,52,53,54], in the inter-disciplinary management of COVID-19 [55,56,57].

## 5. Conclusions

It is well known that multiple viral pathogens, first of all, Herpes and human Papilloma viruses, are directly responsible for the genesis of benign, potentially malignant and malignant lesions of the oral mucosa, underlining the necessity to examine the potential causative role of SARS-CoV-2 in oral lesions.

However, at the current state of knowledge, the small number of cases with oral lesions described in the literature and diagnosed by specialized clinicians, the poor quality of the studies reporting them, along with the heterogeneity of the denominations used, the complexity and polymorphism of the macroscopic pictures delineated, as well as the scarcity of microscopic features and data concerning the timing of appearance, patients’ characteristics, COVID-19 severity and ongoing treatments, do not make it possible to accurately estimate the prevalence of these lesions, their degree of association with COVID-19 severity and their occurrence in the natural history of the disease.

## Figures and Tables

**Table 1:** Data recorded or computed by reported findings based on eligibility criteria: first author, year, journal and online publication date, funding and quality of systematic the reviews included in the present study and listed in alphabetical order; number and design of analyzed studies, sample size, gender ratio and mean age of investigated population; total number or prevalence of cases diagnosed, through clinical exam, with oral lesions among the investigated population; COVID-19 illness severity, classified as mild/moderate or severe/hospitalization and ongoing COVID-19 treatment; macroscopic and microscopic features and related frequency of reported oral lesions.

Source	Analyzed Studies	Cases Diagnosed with Oral Lesions	Macroscopic and Microscopic Features of Reported Primary Oral Lesions
First author, year Journal Online date Funding Meta-Analysis Quality (AMSTAR2)	Number Design **Investigated Population** Sample size (n.) Gender ratio (M/F) Mean age (y.o.)	Number and/or Prevalence (n.—%) Gender ratio (M/F) Mean age (y.o.) COVID-19 Severity COVID-19 Treatment	Number or Prevalence (n.—%) Single/Multiple—Location Cyto/Histopathology **Erosions and Ulcers** Aphthous-like; Erythema Multiforme-like; Herpetiform **Plaques** White; Red **Vesicles and Bullae** **Maculae and Petechiae**
Amorim Dos Santos, 2021 J Dent Res 11 Sept 2020 No funding No meta-analysis Critically low quality	Studies, n.8 OBS (n.0) CS (n.0) CR (n.7) Sample size, n.8 Gender ratio, 5M/3F Mean age, 53.75 y.o.	Cases, n.8—100% Gender ratio, 5M/3F Mean age, 53.75 y.o. COVID-19 severity: Hospitalized, n.3; Non-hospitalized, n.3 MD, n.2 COVID-19 treatment: MD	**Erosions and Ulcers,** n.5—62.5% Apht., n.1—12.5%; EM, n.0—0%; Herp., n.1—12.5% 2Single/3Multiple—Location: tongue, n.3; hard palate, n.1 cheeks, n.1; N/D, n.1 Cyto/Histopathology (Soares et al., 2020): Epithelium—severe vacuolization and occasional exocytosis. Mesenchymal layer—diffuse chronic inflammatory infiltrate (mostly positive for CD3 and CD8), mainly adjacent to salivary glands, focal areas of necrosis and hemorrhage, thrombi in deep small vessels. Insitu hybridization negative for HHV-1, HHV-2, CMV, Treponema Pallidum and EBV; (Ansari et al., 2020): Edema, desquamation, granulation and ulceration with invasion of mononuclear and neutrophilic cells, suggesting a bacterial superinfection. **Plaques**, n.1—12.5% White, n.1—12.5%; Red, n.0—0% Single—Location: tongue Cyto/Histopathology: MD COVID-19 severity: hospitalized **Vesicles and Bullae,** n.1—12.5% Multiple—Location: lip Cyto/Histopathology: MD COVID-19 severity: hospitalized **Maculae and Petechiae,** n.3—37.5% Erythematous Maculae, n.2—25% Multiple—Location: palate, n.2; tongue, n.1; lip, n.1 Petechiae, n.1—12.5% Multiple—Location:palate Cyto/Histopathology: MD **Other lesions described** Nodule, n.1; Desquamative gingivitis
Aragoneses, 2021 Front Med (Lausanne) 25 Aug 2021 No funding Meta-analysis Critically low quality	Studies, n.6 OBS (n.4) CS (n.0) CR (n.2) Sample size n.2762 Gender ratio, MD Mean age, MD	Cases, 33% Gender ratio, MD Mean age, MD COVID-19 severity: MD COVID-19 treatment: MD	**Erosions and Ulcers,** n.163—17.9% Apht., n.35—3.8%; EM, n.1—0.1%; Herp., n.3—0.33% Single/Multiple: MD—Location: MD Cyto/Histopathology: MD **Plaques,** n.0—0% White, n.0—0%; Red, n.0—0% **Vesicles and Bullae,** n.0—0% **Maculae and Petechiae,** n.34—3.7% Erythematous maculae, n.20—2.2% Single/Multiple: MD—Location: MD Cyto/Histopathology: MD Petechiae, n.14 Single/Multiple: MD—Location: MD Cyto/Histopathology: MD
Bhujel, 2021 Br J Oral Maxillofac Surg Nov 2021 No funding No meta-analysis Critically low quality	Studies, n.7 OBS (n.3) CS (n.4) CS (n.0) Sample size, n.255 Gender ratio, 160M/95F Mean age, 56.6 y.o.	Cases, n.210—82.75% Gender ratio, MD Mean age, MD COVID-19 severity: MD COVID-19 treatment: MD	**Erosions and Ulcers,** n.5—2.4% Apht., n.1—0.5%; EM, n.0—0%; Herp., n.1—0.5% Single/Multiple: MD—Location: MD Cyto/Histopathology: MD **Plaques,** n.0—0% White, n.0—0%; Red, n.0—0% **Vesicles and Bullae,** n.19—9.0% Single/Multiple: MD—Location: MD Cyto/Histopathology: MD **Maculae and Petechiae**, n.22—10.5% Erythematous Maculae, n.21—10% Single/Multiple: MD—Location: MD Cyto/Histopathology: MD Petechiae, n.1—0.5% Single/Multiple: MD—Location: MD Cyto/Histopathology: MD Location: tongue, n.34; cheeks, n.33; gingiva, n.13; lip, n.4; palate, n.11; diffuse, n.1 **Other lesions described** Candidiasis, n.28—13.3%; Oral lichen Planus, n.28—13.3%; Hemorrhagic lesions, n.16—7.6%; Desquamative gingivitis, n.1—0.5%; NPD n.7—3.33%
Cuevas-Gonzalez, 2021 Medicine 23 Dec 2021 No funding No meta-analysis Critically low quality	Studies, n.18 OBS (n.10) CS (n.8) CR (n.0) Sample size, n.756 Gender ratio, MD Mean age, MD	Cases, n.10—1.32% Gender ratio, MD Mean age, MD COVID-19 severity: Hospitalization COVID-19 treatment: MD	**Erosions and Ulcers,** n.7—70.0% Apht., n.0—0%; EM, n.0—0%; Herp., n.1—10% Single/Multiple: MD—Location: MD Cyto/Histopathology: MD **Plaques** n.1—10% White, n.1—10%; Red, n.0—0% Single/Multiple: MD—Location: MD Cyto/Histopathology: MD **Vesicles and Bullae,** n.0—0% Single/Multiple: MD—Location: MD Cyto/Histopathology: MD **Maculae and Petechiae,** n.5—50% Erythematous Maculae, n.5—50% Single/Multiple: MD—Location: MD Cyto/Histopathology: MD Petechiae, n.0—0% **Other lesions described** Candidiasis, n.3—30%; Thrombi, n.1—10%
Doceda, 2022 Oral Health Prev Dent 27 Apr 2022 No funding No meta-analysis Low quality	Studies, n.6 OBS (n.3) CS (n.3) CR (n.0) Sample size, n.1606 Gender ratio, 535M/375F Mean age, MD	Cases, n.277—42.21% Gender ratio, MD Mean age, MD COVID-19 severity: 3Hospitalized/3MD COVID-19 treatment: MD	**Erosions and Ulcers,** n.117—42.2% Apht., n.48—17.3%; EM, n.0—0%; Herp., n.0—0% Single/Multiple: MD—Location: MD Cyto/Histopathology: MD **Plaques,** n.0—0% White, n.0—0%; Red, n.0—0% **Vesicles and Bullae,** n.5—1.8% Single/Multiple: MD—Location: MD Cyto/Histopathology: MD **Maculae and Petechiae,** n.45—16.2% Erythematous Maculae, n.30—10.8% Single/Multiple: MD—Location: MD Cyto/Histopathology: MD Petechiae, n.15—5.4% Single/Multiple: MD—Location: MD Cyto/Histopathology: MD Location: tongue, n.3; lip, n.3; palate, n.6 **Other lesions described** Candidiasis, n.32—11.5%; Oral lichen Planus, n.12—4.33%; Hemorrhagic lesions, n.3—1.1%; NPD, n.6—2.17%
Erbaş, 2021 Int J Dermatol 22 Sept 2021 No funding No meta-analysis Critically low quality	Studies, n.19 OBS (n.0) CS (n.4) CR (n.15) Sample size, n.23 Gender ratio, MD Mean age, MD	Cases, n.23—100% Gender ratio, MD Mean age, MD COVID-19 severity: MD COVID-19 treatment	**Erosions and Ulcers,** n.23—104.4% Apht., n.14—60.9%; EM, n.2—8.7%; Herp., n.0—0% Multiple—Location: MD Cyto/Histopathology: MD **Plaques,** n.0—0% White, n.0—0%; Red, n.0—0% **Vesicles and Bullae,** n.2—8.7% Multiple—Location: MD Cyto/Histopathology: MD **Maculae and Petechiae,** n.7—30.4% Erythematous Maculae n.6—26% Single/Multiple: MD—Location: MD Cyto/Histopathology: MD Petechiae, n.1—4.3% Multiple—Location: MD Cyto/Histopathology: MD Location:tongue, n.7; cheeks, n.3; gingiva, n.1; lip, n.6; palate, n.6; diffuse, n.5 **Other lesions described** Tongue depapillation, n.2—8.6%; Hemorrhagic lesions, n.3—12.9% Multiple
Nijakowski, 2022 J Clin Med 14 Apr 2022 No funding Meta-analysis Low quality	Studies, n.12 OBS (n.10) CS (n.2) CR (n.0) Sample size, n.3287 Gender ratio, 1997M/920F (6792MD) Mean age, 50.5 y.o.	Cases, n.1587—13.54% Gender ratio, MD Mean age, MD COVID-19 severity: MD COVID-19 treatment: MD	**Erosions and Ulcers,** n.347—49.6% Apht., n.266—16.8%; EM, n.30—1.9%; Herp., n.17—1.1%; Multiple Cyto/Histopathology: MD **Plaques,** n.4—0.25% White, n.4—0.25%; Red, n.0—0% Single/Multiple: MD Cyto/Histopathology: MD **Vesicles and Bullae,** n.79—4.97% Multiple Cyto/Histopathology: MD **Maculae and Petechiae,** n.261—16.44% Erythematous Maculae, n.198—12.47% Single/Multiple: MD Cyto/Histopathology: MD Petechiae, n.63—3.96% Single/Multiple: MD Cyto/Histopathology: MD Location:tongue, n.295; cheeks and lip, n.218; gingiva, n.45; palate, n.43; diffuse n.37 **Other lesions described** Candidiasis, n.161—10.1%; Oral lichen Planus, n.67—4.2%; Hemorrhagic lesions, n.36—2.3%; Desquamative gingivitis, n.10—0.6%; NPD n.20—1.3%
Orilisi, 2021 Int J Environm Res Public Health 27 Nov 2021 No funding No meta-analysis Low quality	Studies, n.36 OBS (n.8) CS (n.15) CR (n.13) Sample size, n.1219 Gender ratio, 456M/374F and 389 N/D Mean age, 50.8 y.o.	Cases, n.834—68.41% Gender ratio, 423M/380F Mean age, 55.7 y.o. COVID-19 severity. Hospitalized/Non-hospitalized COVID-19 treatment: MD	**Erosions and Ulcers,** n.76—9.11% Apht., n.10—1.2%; EM, n.0—0%; Herp., n.0—0% Single/Multiple: MD—Location: MD Cyto/Histopathology: MD **Plaques,** n.1—1.2% White, n.1—1.2%; Red, n.0—0% Single/Multiple: MD—Location: MD Cyto/Histopathology: MD **Vesicles and Bullae,** n.19—2.3% Single/Multiple: MD—Location: MD Cyto/Histopathology: MD **Maculae and Petechiae,** n.18—2.15% Erythematous Maculae n.2—0.2% Single/Multiple: MD—Location: MD Cyto/Histopathology: MD Petechiae n.16—1.9% Single/Multiple: MD—Location: MD Cyto/Histopathology: MD **Other lesions described** Candidiasis, n.5—0.6%; Swelling of the tongue, n.2—0.2%; NPD, n.7—0.8%
Qi, 2022 AHR 5 Mar 2022 Partial funding from national Institutes of Health (NIH)/National Institute of Dental and Craniofacial Research (NIDCR) U01 DE027512-01 and NIH/National Institute of Aging (NIA) R56 AG067619-01 Meta-analysis Critically low quality	Studies, n.6 OBS (n.6) CS (n.0) CR (n.0) Sample size, n.1562 Gender ratio, MD Mean age, MD	Cases, n.250–16.00% Gender ratio, MD Mean age, MD COVID-19 severity: Hospitalized/Non-hospitalized COVID-19 treatment: MD	**Erosions and Ulcers,** n.77—30.8% Apht.-like, n.61—24.4%; EM, n.26—10.4%; Herp., n.0—0%; Single/Multiple: MD—Location: MD Cyto/Histopathology: MD **Plaques,** n.0—0% White, n.0—0%; Red, n.0—0% **Vesicles and Bullae,** n.19—7.6% Single/Multiple: MD—Location: MD Cyto/Histopathology: MD **Maculae and Petechiae** n.36—14.4% Erythematous Maculae n.22—8.8% Single/Multiple: MD—Location: MD Cyto/Histopathology: MD Petechiae n.14—5.6% Single/Multiple: MD—Location: MD Cyto/Histopathology: MD
Sharma, 2022 Rev Med Virol 10 Mar 2022 No funding No meta-analysis Critically low quality	Studies, n.34 OBS (n.21) CS (n.3) CR (n.10) Sample size, n.764 Gender ratio, MD Mean age, MD	Cases, n.69–9.03% Gender ratio, MD Mean age, MD COVID-19 severity: MD COVID-19 treatment: MD	**Erosions and Ulcers,** n.48—69.6% Apht., n.46—67.7%; EM, n.0—0%; Herp., n.2—2.9% Single/Multiple: MD—Location: MD Cyto/Histopathology: MD **Plaques,** n.0—0% White, n.0—0%; Red, n.0—0% **Vesicles and Bullae,** n.1—1.4% Multiple—Location: MD Cyto/Histopathology: MD **Maculae and Petechiae,** n.2—2.9% Erythematous Maculae, n.2—2.9% Single/Multiple: MD—Location: MD Cyto/Histopathology: MD Petechiae, n.0—0% **Other lesions described** Candidiasis, n.7—10.1%
Silveira, 2022 Arch Oral Biol 12 Feb 2022 Partialfunding fromSectorial Commission for Scientific Research (CSIC) 2018,University of the Republic, Uruguay No meta-analysis Critically low quality	Studies, n.4 OBS (n.0) CS (NA) CR (NA) Sample size, n.5 Gender ratio, 3M/2F Mean age, 47.6 y.o.	Cases, n.5–100% Gender ratio, 3M/2F Mean age, 47.6 y.o. COVID-19 severity: MD COVID-19 treatment: Supportive, n.3 Antibiotics, n.2; Antivirals, n.1	**Erosions and Ulcers,** n.5—100% Apht., n.0—0%; EM, n.0—0%; Herp., n.0—0% Single/Multiple: MD Cyto/Histopathology: Epithelial Layer—Vacuolization (100.0%); Exocytosis 5 (100.0%). Mesenchymal Layer—Inflammation (60.0%); Thrombi/microvascular thrombosis (60.0%); Hemorrhage and Necrosis (40.0) **Plaques,** n.0—0% White, n.0—0%; Red, n.0—0% **Vesicles and Bullae,** n.1—20% Multiple Cyto/Histopathology: MD **Maculae and Petechiae,** n.2—40% Erythematous Maculae, n.2—40% Single/Multiple: MD Cyto/Histopathology: MD Petechiae, n.0—0% Location: tongue, n.2; cheeks, n.1; lip, n.2; palate, n.3
Uzêda-E-Silva, 2021 Stomatologija Mar 2022 No funding No meta-analysis Critically low quality	Studies, n.20 OBS (n.0) CS (n.0) CR (n.1) Letters (n.15) Comments (n.2) Sample size, n.16 Gender ratio, MD Mean age, 40.5 y.o.	Cases, n.16—100% Gender ratio, MD Mean age, 40.5 y.o. COVID-19 severity: MD COVID-19 treatment: MD	**Erosions and Ulcers,** n.5—31.2% Apht., n.2—12.5%; EM, n.1—6.2%; Herp., n.2—12.5% Single/Multiple: MD—Location: MD Cyto/Histopathology: MD **Plaques, n.1** White, n.1; Red, n.0—0% Single/Multiple: MD—Location: MD Cyto/Histopathology: MD **Vesicles and Bullae,** n.2—12.5% Multiple—Location: MD Cyto/Histopathology: MD **Maculae and Petechiae,** n.3—18.7% Erythematous Maculae, n.2—12.5% Single/Multiple: MD Cyto/Histopathology: MD Petechiae, n.1—6.5% Single/Multiple: MD Cyto/Histopathology: MD **Other lesions described** NPD, n. 1—6.25%

Abbreviations: Case report, “CS”; case series, “CS”; observational study, “OBS”; males, “M”; females, “F”; years old, “y.o.”; number, “n.”; Ppercentage, “%”; not defined, “N/D”; missing data, “MD”; Aphthous-like, “Apht.”; Erythema Multiforme-like, “EM”; Herpetiform, “Herp.”; Necrotizing Periodontal Disease, “NPD”.

## Data Availability

Retrieved data are available on Scopus, MEDLINE/PubMed and BioMed Central databases, and on the PROSPERO register.
